# Diazepam Premedication in Primary Augmentation Mammoplasty

**Published:** 2020-04-20

**Authors:** Elizabeth A. Lucich, Nicholas S. Adams, Renee L. Barry, Joshua J. Nelson, Joshua P. Kelley, John D. Renucci

**Affiliations:** ^a^Spectrum Health/Michigan State University Plastic Surgery Residency, Grand Rapids; ^b^Henry Ford General Surgery Residency, Detroit, Mich; ^c^Plastic Surgery Associates, PC, Grand Rapids, Mich

**Keywords:** breast augmentation, augmentation mammoplasty, diazepam premedication, narcotic use, pain control, postoperative nausea and vomiting

## Abstract

**Goals/Purpose:** To evaluate the effects of preoperative oral diazepam on the postoperative course of patients undergoing primary augmentation mammoplasty in an outpatient surgical center. **Methods/Technique:** A retrospective review was conducted of 189 patients undergoing primary breast augmentation at an outpatient surgical center from 2012 to 2015. Patients receiving same-day premedication with oral diazepam were compared with a control group without premedication. Patients with combined surgical procedures were excluded with the exception of minor, superficial procedures. Patient demographics, perioperative medication use, operative details, and postoperative numeric pain scale (0-10) scores were collected. **Results/Complications:** Ninety-three patients (49%) were included in the premedication group and 96 (51%) in the control group. Difference in age, body mass index, implant size, and intraoperative opioid use were not statistically significantly different between the treatment and control groups (*P* > .05). No difference was noted in postoperative nausea, emesis, or antiemetic use between the 2 groups. The operative time was slightly longer in the control group (64.5 minutes vs 58.5 minutes, *P* = .006). Immediate postoperative pain (3.6 vs 4.4) and time to discharge (101 minutes vs 110 minutes) were slightly decreased in the premedication group; however, these values did not reach statistical significance. Intraoperative narcotic use was the same between groups, but postoperative narcotic pain medication use was higher in the premedication group (9.68 mg vs 8.26 mg, *P* = .036). Predischarge pain scores (2.87 vs 2.29, *P* = .006) were also noted to be slightly higher in the premedication group. **Conclusions:** Preoperative diazepam administration does not significantly decrease time to discharge in primary breast augmentation mammoplasty. Furthermore, its use may result in increased postoperative narcotic use and higher pain scores at the time of discharge.

Breast augmentation remains one of the most common cosmetic surgical procedures performed in the United States, with 313,735 surgical procedures performed in 2018.^1^ With an increasing focus on patient satisfaction and the popularity of breast augmentation procedures, it is important that measures are taken to improve patient outcomes and satisfaction. This particular procedure can be associated with adverse effects secondary to surgical technique and due to the anesthesia administered. Patients may have significant pain and discomfort postoperatively, especially when submuscular dissection is performed.^2^ Anesthetic side effects include headache, nausea, vomiting, and altered mental status.^3^ In addition, the use of postoperative opioids has been significantly associated with the development of postoperative nausea and vomiting.^4^

Providing adequate pain control is often a difficult task and a single approach is not appropriate for all patients.^3^ In the past, there has been concern that premedication with anxiolytics could delay time to discharge following day surgery but this has been shown to not hold true in a recent Cochrane review.^5^ Recent data showed that higher preoperative doses of diazepam significantly reduced fentanyl requirements, resulting in shorter recovery times, decreased postoperative nausea/vomiting, and elimination of unintended admissions in breast augmentation.^6^ The goal of this study was to determine the effects of preoperative diazepam on the perioperative course of patients undergoing primary augmentation mammoplasty under general anesthesia, with the hypothesis that the use of preoperative diazepam will improve perioperative course.

## METHODS

Following approval by the institutional review board, a retrospective review of 189 consecutive patients undergoing primary augmentation mammoplasty was conducted. All surgical procedures were performed at a single outpatient surgery center between July 2012 and July 2015 by 4 plastic surgeons and 13 anesthesiologists.

Patients undergoing surgery after December 30, 2014, were routinely administered preoperative oral diazepam. This group was compared with patients undergoing surgery before December 30, 2014, who did not receive anxiolytic premedication. Inclusion criterion included female patients 18 years or older undergoing bilateral primary augmentation mammoplasty. Patients undergoing revision augmentation mammoplasty or concurrent procedures in addition to primary augmentation mammoplasty were excluded, with the exception of minor superficial procedures including punch biopsy (n = 1), skin tag removal (n = 1), abdominal scar revision (n = 3), and nipple-areolar complex reduction (n = 3)

All augmentation mammoplasties were performed through an inframammary incision with implant placement in a subpectoral pocket. Local anesthetic was used routinely in all patients in the form of lidocaine and bupivacaine.

Data were collected from the chart including patient demographics, breast cup size, preoperative medications, operative technique, operative time, time to discharge, intraoperative medication use, postoperative pain and antiemetic medications, and early postoperative course. Opioid medications were converted to morphine-equivalents for a standardized evaluation.

Two-tailed *t* tests were used to compare data between the premedication group and the control group. A Pearson correlation was used to determine the relationship between results and implant size. Statistical significance was set at *P* < .05. Statistical analysis was conducted using SPSS software (SPSS Inc, Chicago, Ill).

## RESULTS

A total of 189 patients were included. Ninety-three patients (49%) were included in the premedication group and 96 in the control group. There were no significant differences in demographic data collected (Table 1).

Within the intraoperative data, there were no statistically significant differences in implant size, number of concurrent procedures performed, or intraoperative opioid use (Table 2). There was a statistically significant decrease in operative times for patients receiving premedication. The operative time for patients receiving diazepam was 58.5 minutes; for those who did not receive diazepam, it was 64.5 minutes (*P* < .05).

Among immediate postoperative data collected, there were no statistically significant differences in immediate postoperative pain, nausea, emesis, antiemetic use, or time to discharge (Table 3). There was a statistically significant increase in postoperative opioid use in patients who received diazepam preoperatively (*P* < .05). Similarly, there was a statistically significant increase in the predischarge pain scores in those who received diazepam premedication (*P* < .05).

The data were further analyzed to determine whether there was a correlation between implant size and postoperative pain scores, opioid use, or time to discharge. There were no statistically significant differences identified (Table 4). There were statistically significant results in regard to patients’ preoperative breast size, body mass index, intraoperative narcotics, total narcotics, and postoperative emesis (Table 5).

## DISCUSSION

The use of preoperative oral diazepam in patients undergoing primary breast augmentation did not improve perioperative or immediate postoperative outcomes.

Patients undergoing breast augmentation frequently report pain, with a higher incidence in those women who received submuscular coverage of the implant.^2^ Unfortunately, opioids, the mainstay treatment of pain, are well known to cause postoperative nausea and vomiting, as well as carry the risk of developing dependence. Premedication with benzodiazepines in some studies has demonstrated fewer postoperative side effects, including postoperative nausea, without delaying recovery time.^7^^,^^8^

In this study, we found no benefit of premedication with diazepam. While immediate postoperative pain was similar between the control group and those receiving diazepam premedication, the postoperative narcotic use and predischarge pain scores were significantly higher in the diazepam group. Caumo et al^9^ found a direct correlation between preoperative anxiety and higher postoperative pain. Other studies have found that the quality of recovery after premedication was no different from controls but the perioperative experience was improved.^8^ While our findings of increased postoperative pain and narcotic use may be statistically significant, the difference in pain and narcotic use is likely clinically insignificant.

The mean operative time was found to be approximately 6 minutes longer in the control group (*P* = .006). This could be due to increasing operative efficiency among the surgeons, as the control group underwent augmentation mammoplasty during an earlier time point. However, all surgeons included in the study had a minimum of 4 years in practice following training.

Another interesting finding of this study was the lack of correlation between implant size and postoperative pain or perioperative opioid use. Among patients undergoing breast augmentation, chronic pain was more prevalent in those who underwent submuscular implant placement than in those with subglandular placement.^2^ It would be reasonable to assume that if the muscle was subjected to increased stress with a larger implant, patients would experience more pain. However, our data did not support that trend. Tebbetts^10^ found that meticulous technique and careful dissection of the submuscular pocket were associated with significantly decreased postoperative pain scores. Based on our findings, it is possible that the pain associated with submuscular placement of the implant is related to trauma of the muscle during dissection rather than the stretch necessary to accommodate the device.

Total opioid use was found to be higher in patients with larger preoperative breast size (B or C) than in those with smaller preoperative size (AA or A). In these patients, however, the increased narcotic use was seen intraoperatively rather than postoperatively. In addition, the implant volume and body mass index were found to be significantly higher in patients within the larger breast size group (B or C). These patients may have had more redistribution of medications during anesthesia requiring higher doses for adequate anesthesia. Conversely, these patients did have larger devices placed that could account for the increased opioid use.

This study did not demonstrate any perioperative benefit of diazepam premedication in primary augmentation mammoplasty. Selective use of anxiolytics may provide the most benefit for patients rather than using anxiolytics in all patients.

## Figures and Tables

**Table 1:** Demographic data of patients undergoing primary augmentation mammoplasty*

	Preoperative diazepam	No diazepam	*P*
	(n = 93)	(n = 96)	
Age, y	33.0	35.2	.060
BMI	20.7	20.7	.980
Smokers	14 (15.1%)	9 (9.4%)	.233

*BMI indicates body mass index.

**Table 2:** Intraoperative data of patients receiving preoperative diazepam versus patients who did not receive preoperative diazepam*

	Preoperative diazepam	No diazepam	*P*
	(n = 93)	(n = 96)	
Operative time, min	58.52	64.5	.006^†^
Implant size, cc	364.95	368.96	.655
Simultaneous procedures	3 (3.3%)	5 (5.2%)	.721
Intraoperative opioid use, MME	11.62	10.61	.141

*MME indicates morphine milligram equivalents.

^†^*P* < .05.

**Table 3:** Postoperative recovery of patients receiving diazepam versus those who did not*

	Preoperative diazepam	No diazepam	*P*
	(n = 93)	(n = 96)	
Immediate postoperative pain score (1-10)	3.58	4.39	.059
Time to discharge, min	101.42	109.94	.069
Postoperative opioid use, MME	9.68	8.26	.036^†^
Postoperative nausea	23 (24.7%)	29 (30.2%)	.399
Postoperative emesis	4 (4.3%)	2 (2.1%)	.439
Postoperative antiemetic use	20 (21.5%)	21 (21.9%)	.951
Predischarge pain score (1-10)	2.87	2.29	.006^†^

*MME indicates morphine milligram equivalents.

^†^*P* < .05.

**Table 4:** The effect of implant size on postoperative pain and nausea

	Implant size	
	Pearson correlation	*P*
Immediate postoperative pain score (1-10)	0.014	.850
Predischarge pain score (1-10)	−0.135	.069
Postoperative opioid use	0.127	.082
Time to discharge	−0.047	.520

**Table 5:** The effect of preoperative breast size on narcotic use and postoperative nausea/emesis*

	Sizes AA and A	Sizes B and C	*P*
	(n = 116)	(n = 72)	
Age, mean (SD), y	33.6 (7.2)	34.6 (8.7)	.44
BMI, mean (SD)	20.3 (2.0)	21.5 (2.7)	.001
Implant size, mean (SD), cm^3^	355.6 (61.7)	385.4 (57.0)	<.001
Operation time, mean (SD), min	62.0 (17.8)	60.2 (11.8)	.39
Time to DC, mean (SD), min	101.8 (31.6)	107.4 (38.5)	.31
Intraoperative narcotics, mean (SD)	10.6 (5.0)	12.0 (4.1)	.04
Postoperative narcotics, mean (SD)	8.7 (4.7)	9.3 (4.7)	.42
Total narcotics, mean (SD)	19.3 (6.3)	21.3 (6.0)	.027
Immediate postoperative pain score (0-10), mean (SD)	4.0 (3.0)	3.7 (2.8)	.45
Pre-DC pain (0-10), mean (SD)	2.5 (1.5)	2.4 (1.5)	.49
Postoperative nausea	28.5% (33/116)	26.4% (19/72)	.76
Postoperative emesis	5.2% (6/116)	0.0% (0/72)	.0498
Postoperative antiemetic	20.7% (24/116)	22.2% (16/72)	.80

*BMI indicates body mass index; DC, discharge.
